# Resting Heart Rate and Cardiovascular Outcomes during Intensive and Standard Blood Pressure Reduction: An Analysis from SPRINT Trial

**DOI:** 10.3390/jcm10153264

**Published:** 2021-07-24

**Authors:** Piotr Sobieraj, Maciej Siński, Jacek Lewandowski

**Affiliations:** Department of Internal Medicine, Hypertension and Vascular Diseases, Faculty of Medicine, Medical University of Warsaw, Banacha Str 1a, 02-097 Warsaw, Poland; piotr.sobieraj@wum.edu.pl (P.S.); maciej.sinski@wum.edu.pl (M.S.)

**Keywords:** heart rate, blood pressure reduction, hypertension

## Abstract

The association between elevated resting heart rate (RHR) as a cardiovascular risk factor and lowering of systolic blood pressure (SBP) to currently recommended values remain unknown. Systolic Blood Pressure Intervention Trial (SPRINT) data obtained from the NHLBI were used to describe the relationship between RHR and SBP reduction to <120 mmHg compared to SBP reduction to <140 mmHg. The composite clinical endpoint (CE) was defined as myocardial infarction, acute coronary syndrome, decompensation of heart failure, stroke, or cardiovascular death. Increased RHR was associated with a higher CE risk compared with low RHR in both treatment arms. A more potent increase of risk for CE was observed in subjects who were allocated to the SBP < 120 mmHg treatment goal. A similar effect of intensive and standard blood pressure (BP) reduction (*p* for interaction, 0.826) was observed in subjects with RHR in the 5th quintile (hazard ratio, 0.78, with 95% confidence interval (CI), 0.55–1.11) and in other quintiles of baseline RHR (hazard ratio, 0.75, with 95% CI, 0.62–0.90). Lower in-trial than baseline RHR was associated with reduced CE risk (hazard ratio, 0.80, with 95% CI, 0.66–0.98). We concluded that elevated RHR remains an essential risk factor independent of SBP reduction.

## 1. Introduction

The range of 60–100 b.p.m. is generally considered the normal resting heart rate (RHR). Even in the normal range, higher RHRs were found clinically unfavorable [1]. Increased RHR reflects sympathetic over activity and is related to an increased myocardial oxygen consumption, arterial stiffness and atherosclerotic lesion formation [2]. Elevated RHR often occurs in combination with higher blood pressure (BP). Subjects with higher RHR are at increased risk of arterial hypertension development [3]. Moreover, since the 1940s, increased RHR has consistently been found to be a marker of increased cardiovascular (CV) morbidity and mortality in both healthy and unhealthy populations, including subjects with hypertension [4,5]. An association of RHR with CV mortality was found for both sexes, all ages, and various races [6,7,8]. In normotensive subjects, after adjustment for age and systolic BP (SBP), an RHR higher by 40 b.p.m. was related to nearly twice higher CV risk and 1.5 times higher CV mortality [7]. Data from The Losartan Intervention for Endpoint reduction (LIFE) study showed that, with each increase in in-treatment RHR by 10 b.p.m., the risk of CV death and all-cause mortality is increased by 25% and 27%, respectively [9].

Both elevated RHR and BP mutually increase their unfavorable effect on CV risk [6]. Thus, the management of RHR in subjects treated for hypertension remains a clinical challenge. Recently, SBP targets were shifted from 140 mmHg toward 120 mmHg and adopted to further reduce CV risk [1,10]. The associations between RHR and CV outcomes were not explored in this new reality. Studies on hypertensive populations investigating clinical outcomes according to both RHR and BP intervention are still lacking.

Therefore, this study aimed to analyze the role of RHR in patients allocated to standard (SBP target < 140 mmHg) and intensive (SBP target < 120 mmHg) antihypertensive treatment.

## 2. Materials and Methods

### 2.1. Data Source

Limited and anonymized data of all Systolic Blood Pressure Intervention Trial (SPRINT) participants were used to perform the analysis. SPRINT was a randomized multicenter study that showed that SBP reduction to a target of <120 mmHg is associated with a reduced risk of CV events compared with reduction to a standard treatment goal (SBP < 140 mmHg) [11].

SPRINT study intervention was related to 25% reduction in CV events risk (hazard ratio, 0.75; 95% confidence interval (CI), 0.64–0.89; *p* < 0.0001) [11].

In the SPRINT, only subjects at high CV risk were enrolled (age >50 years, SBP 130–180 mmHg, and history of CV or chronic kidney disease or Framingham Risk Score for 10-year CV risk > 15%).

### 2.2. Clinical Endpoint (CE) Outcome

Composite CE was defined as myocardial infarction, acute coronary syndrome other than myocardial infarction, exacerbation of heart failure, stroke, and CV death event, identical to the original SPRINT. Individual components of the composite endpoint were not analyzed due to the risk of bias [12]. Safety outcome data were not analyzed.

### 2.3. BP and RHR Measurements

During the SPRINT, BP and RHR were measured using an automated office system (Model 907, Omron Healthcare, Kyoto, Japan). Both BP and RHR were measured after 5 min of rest three times in 1-min intervals. The mean SBP and diastolic BP (DBP) and RHR of the three measurements were computed. In our study, we defined baseline RHR as the first available measurement during the trial. For each individual, in-trial RHR was computed as the mean of all available RHR measurements during the trial. Baseline and in-trial SBP and DBP were computed by analogy.

### 2.4. Statistical Methods

This study presents the results of a post hoc analysis of the SPRINT data. The SPRINT dataset was divided according to quintiles of baseline RHR and then analyzed.

Continuous variables are presented as mean followed by standard deviation; discrete variables are presented as number and percentage. For comparison within groups, ANOVA or χ2 tests were used depending on the characteristic of the variables. Kaplan–Meier curves were used to present survival according to the SPRINT treatment arm and RHR change during the trial.

Cox proportional hazard risk models were used in the analysis. To establish the relationship between RHR and the effect of study intervention, the *p*-value for interaction was calculated. To present nonlinear relationships between RHR and hazard ratio, spline curves were used.

Additional analyses were performed in subjects with baseline RHR < and > than 80 b.p.m. Such a threshold was selected because, in the European Society of Cardiology (ESC) guidelines, 80 b.p.m is proposed as the value distinguishing hypertensive subjects with a higher and lower CV risk [1]. This additional analysis is presented in Supplementary Materials.

The analysis was performed using R 3.6.0 (R Foundation for Statistical Computing, Vienna, Austria), a software environment for statistical computing. Survival, survminer, and RMS packages were used.

## 3. Results

### 3.1. General Baseline Characteristics

The mean age of the 9361 study participants was 67.9 ± 9.4 years. In the analyzed population, 3332 (35.6%) were women, and 2947 (31.5%) were black. Prior CV disease was diagnosed in 1877 (20.1%) patients and chronic kidney disease in 2646 (28.3%) patients. The mean baseline SBP/DBP was 139.7 ± 15.6/78.1 ± 11.9 mmHg, and mean baseline RHR was 66.3 ± 11.6 b.p.m. Baseline RHR in both treatment arms was similar (standard arm, 66.3 ± 11.7 b.p.m., vs. intensive arm, 66.2 ± 11.5 b.p.m.; *p* = 0.762).

### 3.2. Group Characteristics Presented According to Quintiles of Baseline RHR

The quintiles of the baseline RHR were calculated (1st quintile < 56 b.p.m.; 2nd, 56–62 b.p.m.; 3rd, 62–68 b.p.m.; 4th, 68–76 b.p.m.; 5th ≥76 b.p.m.) Baseline characteristics by quintiles of baseline RHR of the study participants are presented in Table 1.

### 3.3. Clinical Outcomes during the Study by Quintiles of Baseline RHR

**Figure 1:**
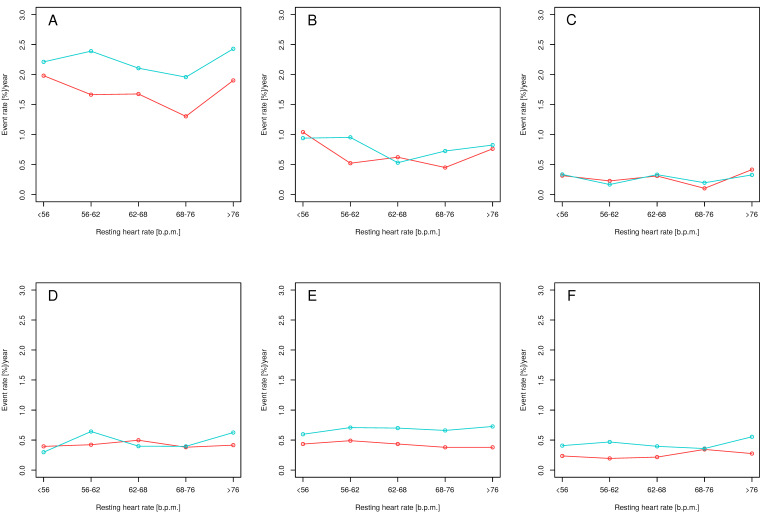
Yearly event rates for mean baseline resting heart rate in intensive (red) and standard (blue) treatment arms for the primary endpoint (**A**), myocardial infarction (**B**), acute coronary syndrome other than myocardial infarction (**C**), stroke (**D**), acute exacerbation of heart failure (**E**), and cardiovascular death (**F**). The primary outcomes were myocardial infarction, acute coronary syndrome other than myocardial infarction, stroke, acute exacerbation of heart failure, and cardiovascular death.

During the trial, 562 (6%) primary endpoint events occurred, including 213 (2.3%) myocardial infarctions, 80 (0.9%) acute coronary syndromes other than myocardial infarction, 132 (1.4%) strokes, 162 (1.7%) exacerbations of heart failure, and 102 (1.1%) CV deaths. Rate of CE and its components by quintile of baseline RHR and treatment arm are presented in Figure 1.

### 3.4. Relationship between Baseline RHR and CE Risk

**Figure 2:**
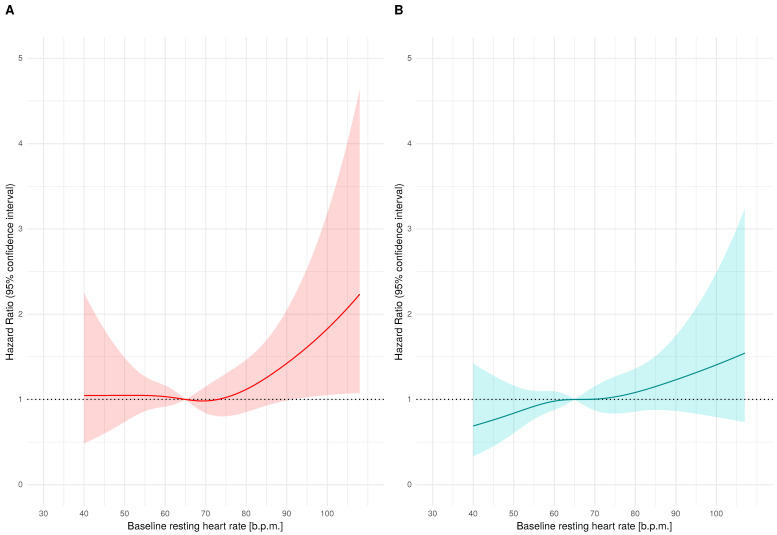
Hazard ratio with 95% confidence intervals against baseline resting heart rate in intensive (panel (**A**), red) and standard (panel (**B**), blue) treatment arms.

Cox proportional hazard risk models estimating hazard ratio for CE were created separately for intensive and standard treatment arms. Both models included baseline RHR, age, sex, baseline and in-trial SBP and DBP, body mass index, estimated glomerular filtration rate, total cholesterol level, serum glucose level, current smoking status, and black race. The hazard ratio against baseline RHR was plotted using cubic splines separately for intensive and standard treatment arms (Figure 2) and assuming risk at 65 b.p.m. to be equal to 1.

In both treatment arms, participants with a higher baseline HR were at higher risk of CE (Figure 2). Comparing the shape of the hazard ratio curves plotted in the intensive and standard treatment arms, we observed that the increase in hazard ratio toward higher RHR values was more potent in subjects allocated to the intensive treatment arm than in those to the standard treatment arm (Figure 2). Using Cox models developed for the hazard ratios plots, assuming HR is equal to 1.0 at 65 b.p.m., we predicted hazard ratios for RHR values in the range 60–100 b.p.m. (with 1-b.p.m. step) separately for intensive and standard treatment arms. The mean predicted hazard ratio was higher in subjects with intensive treatment than those with standard treatment (1.27 ± 0.35 vs. 1.08 ± 0.22, *p* < 0.001), proving a more potent increase of risk in subjects allocated to the intensive treatment arm.

### 3.5. Interaction between Baseline RHR with SBP Reduction

**Figure 3:**
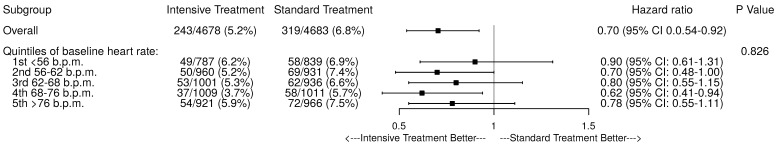
Benefit of intensive versus standard reduction of systolic blood pressure according to quintiles of baseline resting heart rate.

The effect of intensive reduction of SBP was compared in the 5th quintile of baseline RHR (hazard ratio, 0.78, with 95% CI, 0.55–1.11, *p* = 0.174) versus four other quintiles (hazard ratio, 0.75, with 95% CI, 0.62–0.90, *p* = 0.003). The effects of the study intervention according to RHR are presented in Figure 3. No evidence was found for interaction of intensive SBP reduction and baseline HR for CE (*p* for interaction, 0.826).

Among 1877 subjects with prior CVD, the effect of intensive reduction of SBP was compared in the 5th quintile of RHR (hazard ratio, 1.02, with 95% CI, 0.54–1.95, *p* = 0.95) versus the other four quintiles (hazard ratio, 0.80, with 95% CI, 0.59–1.08, *p* = 0.14). There was no interaction between intensive SBP reduction and baseline RHR for primary endpoint events (*p* for interaction, 0.51).

### 3.6. Effect of RHR Reduction on CE Risk

**Figure 4:**
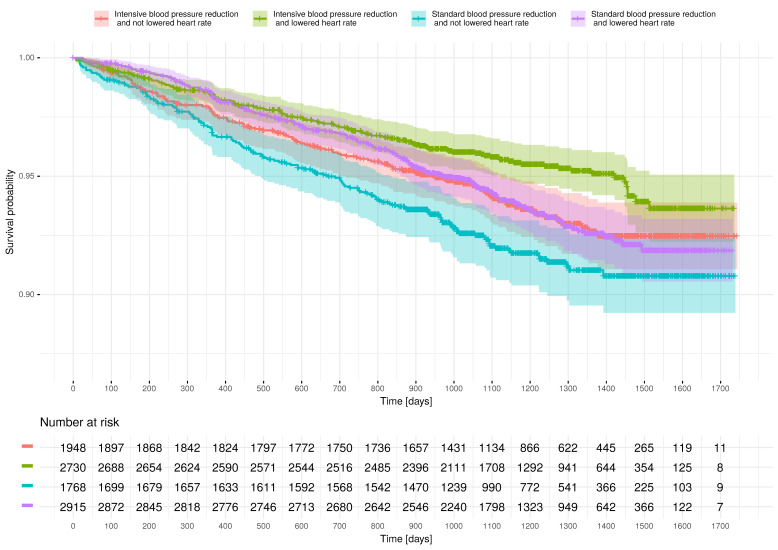
Kaplan–Meier curves presenting clinical-endpoint-free survival according to the study intervention arm and in-trial heart rate in relation to baseline heart rate. In subjects allocated to the intensive treatment arm, survival was highlighted using red (if in-trial heart rate was not lower than that at baseline) or green (if in-trial heart rate was lower than that at baseline) color. In subjects allocated to the standard treatment arm, survival was highlighted using blue (if in-trial heart rate was not lower than that at baseline) or violet (if in-trial heart rate was lower than that at baseline) color.

The mean difference between in-trial and baseline RHRs was 1.6 ± 7.6 b.p.m. In 5645 (60.3%) patients, in-trial RHR was lower than at baseline. Figure 4 presents survival according to trial intervention arm and reduction in RHR. After adjustment for age, sex, allocation to study intervention arm, history of CV and chronic kidney disease, and current smoking status, in-trial RHR lower than baseline RHR was associated with reduced CE risk (hazard ratio, 0.80, with 95% CI, 0.66–0.98) (Table 2).

### 3.7. In-Trial RHR, SBP, and DBP Comparisons According to Quintiles of Baseline RHR

**Figure 5:**
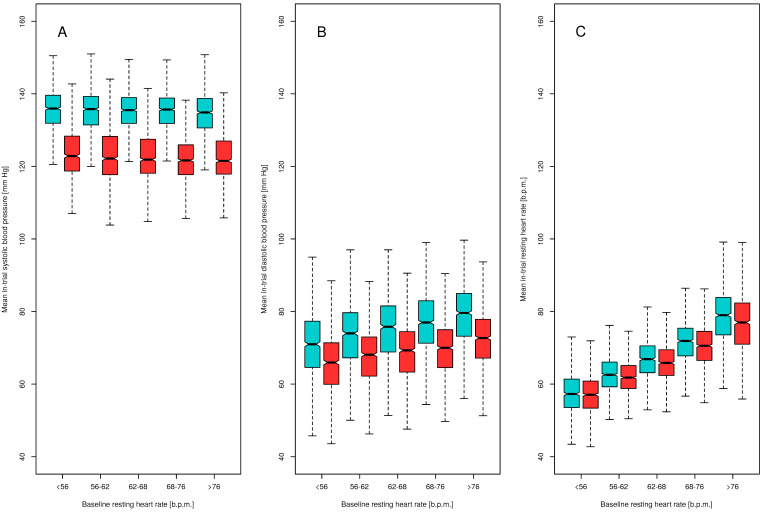
Mean in-trial systolic blood pressure (Panel (**A**)), diastolic blood pressure (Panel (**B**)), and heart rate (Panel (**C**)) according to quintiles of baseline resting heart rate in the intensive (red) and standard (blue) treatment arms.

Boxplots presenting in-trial SBP, DBP, and RHR in quintiles of baseline RHR are presented in Figure 5. In-trial SBP was similar across baseline RHR quintiles. Higher in-trial DBP and higher in-trial RHR were observed in quintiles with higher baseline RHR. Similar findings were found in both treatment arms. In the intensive treatment arm, the in-trial RHR was lower (66.9 ± 9.1 b.p.m.) than that in the standard treatment arm (68 ± 9.6 b.p.m., *p* < 0.001). In all but not in the 1st quintile of baseline RHR, in-trial RHR was higher in the standard treatment arm than that in the intensive treatment arm: 1st quintile, 57.7 ± 6.0 vs. 57.4 ± 5.9 b.p.m., *p* = 0.246; 2nd quintile, 63 ± 5.6 vs. 62.3 ± 5.4 b.p.m., *p* = 0.005; 3rd quintile, 67.1 ± 5.6 vs. 66.1 ± 5.4 b.p.m., *p* < 0.001; 4th quintile, 71.7 ± 6.2 vs. 70.6 ± 6.1 b.p.m., *p* < 0.001; 5th quintile, 78.8 ± 8.0 vs. 76.9 ± 8.5 b.p.m.; *p* < 0.001.

## 4. Discussion

The results of the current study confirms that elevated baseline RHR regardless of BP control contributes to increased CV risk. We showed that increase in CV risk associated with higher RHR was more potent in the intensive than in the standard treatment group. Moreover, we have shown that lower in-trial RHR than at baseline is associated with a lower risk for cardiovascular events.

To date, studies directly assessing the impact of intensive BP reduction on CV risk according to baseline RHR values are still lacking. Previously, some studies found that, in hypertensive subjects receiving antihypertensive therapy, elevated RHR was still responsible for an unfavorable clinical outcome; however, subjects did not reach the target BP values as low as those in the intensively treated group of the SPRINT. Accordingly, in high-risk participants of The Valsartan Antihypertensive Long-term Use Evaluation trial, the adjusted hazard ratio for the first composite event in the highest quintile of baseline RHR compared to pooled lowest quintiles was 1.73, but BP was reduced to 139/79 mmHg and 138/78 mmHg in the two study arms at the end of the trial [13]. Similar to our findings in lower RHR quintiles, there were significantly more primary endpoints in subjects with uncontrolled BP than in those with controlled BP [13]. Similar conclusions were drawn from the International Verapamil-Trandolapril Study (INVEST) trial [14]. In this study, elevated baseline RHR was still associated with adverse outcomes, while BP was controlled approximately in 71% of patients and reduced to 130/76 mmHg.

Most of our remaining results are in line with previous investigations, which consistently have shown that, in hypertensive populations, elevated RHR unfavorably affects CV outcome [7,8,9]. However, the relationship between RHR and CV risk was not described earlier at as low SBP values as currently recommended. Thus, our findings showing that subjects in the intensive treatment group, who achieved SBP values classified as optimal or high normal, still had a higher CV risk when RHR is elevated, should be considered as new. Moreover, in subjects with controlled (BP < 140/90 mmHg) or uncontrolled hypertension (BP > 140/90 mmHg) in the VALUE trial, similar conclusions were drawn, despite these subjects achieving higher BP (130/77 ± 7.9/7.1 mmHg and 153/84 ± 13.3/9.3 mmHg, respectively) [13]. Similar effects of increased RHR were also observed in normotensive subjects with BP values comparable to those achieved in the intensive treatment arm [3,6]. Moreover, we would like to underline that an increase in hazard ratio toward higher RHR values is more potent in subjects allocated to the intensive treatment arm than in the standard treatment group. However, the results presented in Figure 2 should not be used to compare absolute risks related to RHR in subjects with lower and higher BP, but rather to underline that elevated RHR helps to recognize subjects at higher risk, even when BP is well-controlled. Thus, in the era of “lower is better” BP treatment targets in hypertension, RHR should be taken more seriously in the evaluation of CV risk in each patient.

Up-to-date management of elevated CV risk in hypertensive subjects with increased RHR remains unknown. Neither the American Heart Association nor the recent International Society of Hypertension guidelines have proposed management of elevated RHR in subjects with hypertension [10,15]. Despite identification of RHR threshold >80 b.p.m. in ESC guidelines as the risk modifier, the management of CV risk in subjects with elevated RHR was not presented [1]. In the earlier statement prepared by European Society of Hypertension (ESH), RHR reduction using pharmacological methods, along with antihypertensive treatment, was suggested in symptomatic subjects with high RHR [16]. Currently, use of betablockers as the most popular method of RHR lowering may not be reasonable in hypertensive subjects without CV comorbidities, despite the fact that, in subjects with HR >80 b.p.m., marked sympathetic overactivity was found. However, ESC guidelines suggest the use of betablockers when hypertension is associated with sympathetic overactivation and concurrent HR elevation [17]. On the other site, previous studies did not provide sufficient evidence that the use of betablockers with subsequent RHR reduction significantly improves prognosis. In the LIFE study in which atenolol was compared with losartan, subjects treated with atenolol had significantly lower RHR and higher mortality at similar BP reduction [18]. In the INVEST study, subjects were treated with atenolol or verapamil, and although lower RHR was observed in the atenolol group in both treatment strategies, no difference in clinical outcome was found [14]. Moreover, in the Anglo-Scandinavian Cardiac Outcomes Trial (ASCOT) at the final visit, subjects treated with atenolol-based regimen had fewer major CV events than subjects treated with amlodipine, despite similar BP control and lower pulse rate by 11.2 + 12.2 b.p.m. [19]. Some studies showed that a lower RHR may be obtained with interventional procedures, such as renal denervation and carotid baroreceptor stimulation; however, their role still does not extend beyond experimental research [20,21].

Our study (Supplementary Materials) showed that RHR > 80 b.p.m. is associated with a higher hazard ratio for CV outcome in subjects allocated to the intensive treatment arm (hazard ratio, 1.31, with 95% CI, 0.88–1.93) than in subjects allocated to the standard treatment arm (hazard ratio, 1.09, with 95% CI, 0.77–1.52) compared with RHR < 80 b.p.m. This observation supports the ESC recommendation to consider HR > 80 b.p.m. as a CV risk and might be important from a practical point of view. Once the SBP target is reached, the association of high RHR with CV risk is more evident. Therefore, increased RHR as a marker of CV risk should alert physicians, especially when patients respond well to antihypertensive therapy. Consequently, more attention should be paid to the available methods of RHR reduction.

Currently, we also have shown that, independent from the study intervention, subjects with lower in-trial RHR than baseline RHR have better prognosis than subjects with higher in-trial RHR than baseline RHR. Similar observations were also made by other authors [8]. In LIFE study, persistence or development of HR ≥ 84 b.p.m. was associated with an 89% greater risk of CV death and a 97% increased risk of all-cause mortality [9]. The results were consistent and independent of BP decrease, randomized treatment assignment, and other risk factors. Moreover, Paul et al. showed that subjects with a low baseline and in-trial RHR compared to subjects with low baseline and high in-trial RHR had a 52% increase in the risk of all-cause mortality [8]. CV mortality showed a similar pattern with an increase by 91%. Interestingly, rate-lowering therapy did not have an independent effect on outcomes in their analysis.

The main limitation of our study is that both baseline and in-trial RHRs were measured using automated office BP measurement (AOBPM). Previous differences were found between the pulse rate assessed by the physician and the heart rate obtained via electrocardiography [22]. The method of RHR estimation in the current study was not verified against other methods. However, in previous studies performed in the hypertensive population, RHR was evaluated using ambulatory BP monitoring, which is closest to the AOBPM method of BP estimation [23,24]. During the SPRINT trial, there was not a consistently used attended (with observer) or unattended (without observer) AOBPM. [25] About 40% of the measurements were made unattended. We cannot predict how this affected our results. The other important limitation of our study is its post hoc characteristic. We also did not include adjustment of heart rate-limiting therapy and antihypertensive drugs in our analysis. The analysis was not performed due to the fact that some participants could receive such therapy only temporarily as antihypertensive therapy (e.g., betablockers) [26]. Similarly, SPRINT trial protocol assumed also down-titration of the antihypertensive agents when the SBP goal was achieved in the standard treatment arm [11]. According to the SPRINT study regimen, the use of beta-adrenergic agents was encouraged in participants with coronary artery disease [26].

## 5. Conclusions

In conclusion, it should be noted that, although CV risk reduction follows intensive SBP decrease in many hypertensive patients, residual risk at least in part related to an increased RHR remains. Proven benefits of intensive BP reduction along RHR spectrum remains in line with current recommendations [1]. Elevated RHR should be used to recognize higher CV risk, especially in subjects with well-controlled BP. Therefore, no physician should ignore an increased RHR, even at optimal BP control. Our findings provide suggestions that a reduction of RHR using antihypertensive drugs may be beneficial in subjects with hypertension and tachycardia; however, no studies investigated that issue in a such special population. Moreover, the most obvious choice could be betablockers. Up to now, however, there are no studies comparing betablockers with other drugs in hypertensives with tachycardia and further studies are needed.

The unresolved problem of how to manage hypertensive patients with an increased RHR and whether the RHR >80 b.p.m. recommended by ESC guidelines actually reflects the upper limit of the RHR at which CV risk increases remains. This issue warrants further clinical investigations.

## Figures and Tables

**Table 1:** Characteristic study population according to baseline resting heart rate quintile.

	1st Quintile RHR <56 b.p.m. *n* = 1626	2nd Quintile RHR 56–62 b.p.m. *n* = 1891	3rd Quintile RHR 62–68 b.p.m. *n* = 1937	4th Quintile RHR 68–76 b.p.m. *n* = 2020	5th Quintile RHR >76 b.p.m. *n* = 1887	*p*
Age (years)	70.6 ± 8.8	69.3 ± 9.3	68 ± 9.2	66.8 ± 9.3	65.3 ± 9.5	<0.001
Female sex (*n*, %)	465 (28.6)	624 (33)	704 (36.3)	796 (39.4)	743 (39.4)	<0.001
Current smoking status (*n*, %)	116 (7.1)	168 (8.9)	222 (11.5)	306 (15.1)	428 (22.7)	<0.001
Black race (*n*, %)	400 (24.6)	491 (26)	589 (30.4)	677 (33.5)	790 (41.9)	<0.001
BMI (kg/m^2^)	29.3 ± 5.4	29.5 ± 5.5	29.7 ± 5.8	30.2 ± 5.9	30.4 ± 6.1	<0.001
History of cardiovascular disease (*n*, %)	454 (27.9)	449 (23.7)	364 (18.8)	335 (16.6)	275 (14.6)	<0.001
History of clinical cardiovascular disease (*n*, %)	397 (24.4)	384 (20.3)	304 (15.7)	260 (12.9)	217 (11.5)	<0.001
History of subclinical cardiovascular disease (*n*, %)	100 (6.2)	102 (5.4)	97 (5)	111 (5.5)	83 (4.4)	0.205
History of chronic kidney disease (*n*, %)	558 (34.3)	555 (29.3)	561 (29)	517 (25.6)	455 (24.1)	<0.001
Baseline SBP (mmHg)	141.7 ± 16.2	140.1 ± 15.9	139.2 ± 15.1	139.5 ± 15.2	138.2 ± 15.4	<0.001
Baseline DBP (mmHg)	72.9 ± 11.6	75.9 ± 11.2	77.9 ± 11.4	80.2 ± 11.5	82.9 ± 11.7	<0.001
Baseline RHR (b.p.m.)	51 ± 3.6	58.6 ± 1.7	64.4 ± 1.7	71.3 ± 2.3	83.5 ± 7.3	<0.001
In-trial SBP (mmHg)	130.3 ± 9.8	129.6 ± 9.9	129.2 ± 9.8	129.3 ± 10.1	128.9 ± 9.7	<0.001
In-trial DBP (mmHg)	68.4 ± 9	70.4 ± 8.9	72 ± 9	73.5 ± 8.9	75.8 ± 9	<0.001
In-trial RHR (b.p.m.)	57.6 ± 6	62.6 ± 5.5	66.6 ± 5.5	71.1 ± 6.2	77.9 ± 8.3	<0.001
Allocation to intensive treatment arm (*n*, %)	787 (48.4)	960 (50.8)	1001 (51.7)	1009 (50)	921 (48.8)	0.252
EGFR (mL/min/m^2^)	68 ± 18.7	70.3 ± 19.6	71.3 ± 20.5	73.6 ± 21.2	74.8 ± 21.9	<0.001
Serum creatinine (mg/dL)	1.1 ± 0.3	1.1 ± 0.3	1.1 ± 0.3	1 ± 0.3	1.1 ± 0.3	<0.001
Glucose (mg/dL)	97.9 ± 11.2	98 ± 12.2	98.1 ± 12	99.5 ± 14.6	100.4 ± 16.5	<0.001
Total cholesterol (mg/dL)	180.5 ± 38.1	183.7 ± 38.7	190.7 ± 40.8	195.5 ± 41.3	198.6 ± 43.5	<0.001
HDL (mg/dL)	51.9 ± 13	52.3 ± 13.9	53 ± 14.1	53.4 ± 15.1	53.6 ± 15.8	<0.001
Triglicerydes (md/dL)	116.5 ± 69.8	118.4 ± 65.8	122.3 ± 72	132.9 ± 97.6	137.9 ± 127.6	<0.001
On aspirin (*n*, %)	971 (59.9)	1037 (55.1)	1041 (54)	930 (46.2)	777 (41.2)	<0.001
On statin (*n*, %)	826 (51.2)	889 (47.3)	855 (44.4)	778 (38.9)	706 (37.8)	<0.001

BMI, body mass index; SBP, systolic blood pressure; DBP, diastolic blood pressure; RHR, resting heart rate; EGFR, estimated glomerular filtration rate; HDL, high density lipoprotein.

**Table 2:** Proportional hazard risk model evaluating impact of lower in-trial than baseline RHR.

Parameter	Hazard Ratio	95% Confidence Interval	*p*-Value
Age (years)	1.05	1.04–1.06	<0.001
Female sex	0.69	0.57–0.84	<0.001
Allocation to intensive treatment arm	0.74	0.63–0.87	<0.001
Current smoking status	1.92	1.51–2.45	<0.001
History of cardiovascular disease	2.17	1.81–2.599	<0.001
History of chronic kidney disease	1.46	1.22–1.74	<0.001
Baseline SBP (mm Hg)	1.01	1.00–1.01	0.024
Baseline RHR (b.p.m.)	1.01	1.00–1.01	0.190
Total serum cholesterol (mg/dl)	1.00	0.99–1.00	0.027
Lower in-trial RHR than at baseline	0.80	0.66–0.98	0.020

SBP, systolic blood pressure; RHR, resting heart rate.

## Data Availability

SPRINT trial data (accession number HLB02021921a) were obtained via the National Heart, Lungs and Blood Institute (NHLBI) Biologic Specimen and Data Repository Information Coordinating Center. The data are available upon reasonable request from NHLBI. The authors have no right to share the data. This manuscript does not necessarily reflect the opinions or views of the SPRINT Research Group or the NHLBI.

## Supplementary Materials

Supplementary analyses were performed using RHR of 80 b.p.m. as threshold, according to the ESC Guidelines on Hypertension. The results show similar conclusions as presented above and are presented in a supplementary file online. The following are available online at https://www.mdpi.com/article/10.3390/jcm10153264/s1, Supplementary Analysis: The results of the analysis with threshold value RHR = 80 b.p.m in all SPRINT participants and in subjects with prior cardiovascular disease.

## References

[1] Williams B., Mancia G., Spiering W., Agabiti Rosei E., Azizi M., Burnier M., Clement D.L., Coca A., De Simone G., Dominiczak A. (2018). 2018 ESC/ESH Guidelines for the management of arterial hypertension. Eur. Heart J..

[2] Custodis F., Schirmer S.H., Baumhakel M., Heusch G., Bohm M., Laufs U. (2010). Vascular pathophysiology in response to increased heart rate. J. Am. Coll. Cardiol..

[3] Palatini P., Dorigatti F., Zaetta V., Mormino P., Mazzer A., Bortolazzi A., D’Este D., Pegoraro F., Milani L., Mos L. (2006). Heart rate as a predictor of development of sustained hypertension in subjects screened for stage 1 hypertension: The HARVEST Study. J. Hypertens..

[4] Levy R.L., White P.D., Stroud W.D., Hillman C.C. (1946). Transient tachycardia; prognostic significance alone and in association with transient hypertension. J. Am. Med Assoc..

[5] Zhong C., Zhong X., Xu T., Peng H., Li H., Zhang M., Wang A., Xu T., Sun Y., Zhang Y. (2015). Combined effects of hypertension and heart rate on the risk of stroke and coronary heart disease: A population-based prospective cohort study among Inner Mongolians in China. Hypertens. Res..

[6] Benetos A., Rudnichi A., Thomas F., Safar M., Guize L. (1999). Influence of heart rate on mortality in a French population: Role of age, gender, and blood pressure. Hypertension.

[7] Gillman M.W., Kannel W.B., Belanger A., D′Agostino R.B. (1993). Influence of heart rate on mortality among persons with hypertension: The Framingham Study. Am. Heart J..

[8] Paul L., Hastie C.E., Li W.S., Harrow C., Muir S., Connell J.M., Dominiczak A.F., McInnes G.T., Padmanabhan S. (2010). Resting heart rate pattern during follow-up and mortality in hypertensive patients. Hypertension.

[9] Okin P.M., Kjeldsen S.E., Julius S., Hille D.A., Dahlöf B., Edelman J.M., Devereux R.B. (2010). All-cause and cardiovascular mortality in relation to changing heart rate during treatment of hypertensive patients with electrocardiographic left ventricular hypertrophy. Eur. Heart J..

[10] Whelton P.K., Carey R.M., Aronow W.S., Casey D.E., Collins K.J., Himmelfarb C.D., DePalma S.M., Gidding S., Jamerson K.A., Jones D.W. (2018). 2017 ACC/AHA/AAPA/ABC/ACPM/AGS/APhA/ASH/ASPC/NMA/PCNA Guideline for the Prevention, Detection, Evaluation, and Management of High Blood Pressure in Adults: Executive Summary: A Report of the American College of Cardiology/American Heart Association Task Force on Clinical Practice Guidelines. Hypertension.

[11] Wright J.T., Whelton P.K., Reboussin D.M. (2016). A Randomized Trial of Intensive versus Standard Blood-Pressure Control. N. Engl. J. Med..

[12] Freemantle N. (2001). Interpreting the results of secondary end points and subgroup analyses in clinical trials: Should we lock the crazy aunt in the attic?. BMJ.

[13] Julius S., Palatini P., Kjeldsen S.E., Zanchetti A., Weber M.A., McInnes G.T., Brunner H.R., Mancia G., Schork M.A., Hua T.A. (2012). Usefulness of heart rate to predict cardiac events in treated patients with high-risk systemic hypertension. Am. J. Cardiol..

[14] Kolloch R., Legler U.F., Champion A., Cooper-DeHoff R.M., Handberg E., Zhou Q., Pepine C.J. (2008). Impact of resting heart rate on outcomes in hypertensive patients with coronary artery disease: Findings from the INternational VErapamil-SR/trandolapril STudy (INVEST). Eur. Heart J..

[15] Unger T., Borghi C., Charchar F., Khan N.A., Poulter N.R., Prabhakaran D., Ramirez A., Schlaich M., Stergiou G.S., Tomaszewski M. (2020). 2020 International Society of Hypertension global hypertension practice guidelines. J. Hypertens..

[16] Palatini P., Rosei E.A., Casiglia E., Chalmers J., Ferrari R., Grassi G., Inoue T., Jelakovic B., Jensen M.T., Julius S. (2016). Management of the hypertensive patient with elevated heart rate. J. Hypertens..

[17] Fisher J.P., Paton J.F.R. (2011). The sympathetic nervous system and blood pressure in humans: Implications for hypertension. J. Hum. Hypertens..

[18] Dahlöf B., Devereux R.B., Kjeldsen S.E., Julius S., Beevers G., de Faire U., Fyhrquist F., Ibsen H., Kristiansson K., Lederballe-Pedersen O. (2002). Cardiovascular morbidity and mortality in the Losartan Intervention For Endpoint reduction in hypertension study (LIFE): A randomised trial against atenolol. Lancet.

[19] Dahlöf B., Sever P.S., Poulter N.R., Wedel H., Beevers D.G., Caulfield M., Collins R., Kjeldsen S.E., Kristinsson A., McInnes G.T. (2005). Prevention of cardiovascular events with an antihypertensive regimen of amlodipine adding perindopril as required versus atenolol adding bendroflumethiazide as required, in the Anglo-Scandinavian Cardiac Outcomes Trial-Blood Pressure Lowering Arm (ASCOT-BPLA): A multicentre randomised controlled trial. Lancet.

[20] Böhm M., Kario K., Kandzari D.E., Mahfoud F., Weber M.A., Schmieder R.E., Tsioufis K., Pocock S., Konstantinidis D., Choi J.W. (2020). Efficacy of catheter-based renal denervation in the absence of antihypertensive medications (SPYRAL HTN-OFF MED Pivotal): A multicentre, randomised, sham-controlled trial. Lancet.

[21] Seravalle G., Dell’Oro R., Grassi G. (2019). Baroreflex activation therapy systems: Current status and future prospects. Expert Rev. Med. Devices.

[22] Oba Y., Hoshide S., Kabutoya T., Kario K. (2018). Increased Resting Heart Rate on Electrocardiogram Relative to In-office Pulse Rate Indicates Cardiac Overload: The J-HOP Study. Am. J. Hypertens..

[23] Palatini P., Thijs L., Staessen J.A., Fagard R.H., Bulpitt C.J., Clement D.L., De Leeuw P.W., Jaaskivi M., Leonetti G., Nachev C. (2002). Predictive Value of Clinic and Ambulatory Heart Rate for Mortality in Elderly Subjects With Systolic Hypertension. Arch. Intern. Med..

[24] Roerecke M., Kaczorowski J., Myers M.G. (2019). Comparing Automated Office Blood Pressure Readings With Other Methods of Blood Pressure Measurement for Identifying Patients With Possible Hypertension: A Systematic Review and Meta-analysis. JAMA Intern. Med..

[25] Johnson K.C., Whelton P.K., Cushman W.C., Cutler J.A., Evans G.W., Snyder J.K., Ambrosius W.T., Beddhu S., Cheung A.K., Fine L.J. (2018). Blood Pressure Measurement in SPRINT (Systolic Blood Pressure Intervention Trial). Hypertension.

[26] Ambrosius W.T., Sink K., Foy C.G., Berlowitz D., Cheung A.K., Cushman W.C., Fine L.J., Goff J.D.C., Johnson K.C., Killeen A.A. (2014). The design and rationale of a multicenter clinical trial comparing two strategies for control of systolic blood pressure: The Systolic Blood Pressure Intervention Trial (SPRINT). Clin. Trials.

