# Supplementary material for: Resting Heart Rate and Cardiovascular Outcomes during Intensive and Standard Blood Pressure Reduction: An Analysis from SPRINT Trial
Source: J Clin Med. 2021 Jul 24;10(15):3264. doi: 10.3390/jcm10153264 (PMC8347499; doi:10.3390/jcm10153264)
Supplement: Supplementary file 1 [file jcm-10-03264-s001.zip › jcm-1299493-supplementary.pdf]

The results of the analysis with threshold value RHR = 80 b.p.m in all SPRINT participants and in subjects with prior cardiovascular disease.

After the adjustment for age, sex, race, baseline and in-trial SBP and DBP, BMI, GFR, cholesterol, glucose concentration, current smoking status in subjects allocated to standard treatment group, RHR > 80 b.p.m. was associated with increased by 9% risk (hazard ratio 1.09 with 95% CI 0.77–1.52) in comparison to subjects with RHR < 80 b.p.m. In subjects allocated to intensive treatment arm RHR > 80 b.p.m. was associated with increased by 31% risk (hazard ratio 1.31 with 95% CI 0.88–1.93) in comparison to subjects with RHR < 80 b.p.m.

When the effect of intensive lowering of SBP was compared in subjects with baseline HR≥80 b.p.m. (hazard ratio 0.79 with 95% CI 0.51–1.24, $p = 0.311$) versus subjects with baseline HR<80 b.p.m., (hazard ratio 0.75 with 95% CI 0.62–0.89, $p = 0.002$), no evidence was found for interaction for intensive SBP lowering and baseline HR for primary endpoint event (p for interaction 0.819).

Subanalysis in subjects with the history of CVD

When effect of intensive lowering of SBP was compared in subjects with baseline HR ≥ 80 b.p.m. (hazard ratio 0.92 with 95% CI 0.37–2.27, $p = 0.86$) versus subjects with baseline HR < 80 b.p.m., (hazard ratio 0.82 with 95% CI 0.62–1.09, $p = 0.18$), no evidence was found for interaction for intensive SBP lowering and baseline HR for primary endpoint event (p for interaction 0.81).
